# Structural and antimicrobial properties of human pre-elafin/trappin-2 and derived peptides against *Pseudomonas aeruginosa*

**DOI:** 10.1186/1471-2180-10-253

**Published:** 2010-10-08

**Authors:** Audrey Bellemare, Nathalie Vernoux, Sébastien Morin, Stéphane M Gagné, Yves Bourbonnais

**Affiliations:** 1 Département de biochimie, microbiologie et bio-informatique, Institut de biologie intégrative et des systèmes and Regroupement PROTEO, Université Laval, Québec, Qc, Canada; 2 INAF, Département des sciences des aliments et de nutrition Faculté des sciences de l'agriculture et de l'alimentation Université Laval. Québec, Qc, Canada; 3 Division of Structural Biology, Biozentrum, University of Basel, Klingelbergstrasse 70, CH-4056 Basel, Switzerland

## Abstract

**Background:**

Pre-elafin/trappin-2 is a human innate defense molecule initially described as a potent inhibitor of neutrophil elastase. The full-length protein as well as the N-terminal "cementoin" and C-terminal "elafin" domains were also shown to possess broad antimicrobial activity, namely against the opportunistic pathogen *P. aeruginosa*. The mode of action of these peptides has, however, yet to be fully elucidated. Both domains of pre-elafin/trappin-2 are polycationic, but only the structure of the elafin domain is currently known. The aim of the present study was to determine the secondary structures of the cementoin domain and to characterize the antibacterial properties of these peptides against *P. aeruginosa*.

**Results:**

We show here that the cementoin domain adopts an α-helical conformation both by circular dichroism and nuclear magnetic resonance analyses in the presence of membrane mimetics, a characteristic shared with a large number of linear polycationic antimicrobial peptides. However, pre-elafin/trappin-2 and its domains display only weak lytic properties, as assessed by scanning electron micrography, outer and inner membrane depolarization studies with *P. aeruginosa *and leakage of liposome-entrapped calcein. Confocal microscopy of fluorescein-labeled pre-elafin/trappin-2 suggests that this protein possesses the ability to translocate across membranes. This correlates with the finding that pre-elafin/trappin-2 and elafin bind to DNA *in vitro *and attenuate the expression of some *P. aeruginosa *virulence factors, namely the biofilm formation and the secretion of pyoverdine.

**Conclusions:**

The N-terminal cementoin domain adopts α-helical secondary structures in a membrane mimetic environment, which is common in antimicrobial peptides. However, unlike numerous linear polycationic antimicrobial peptides, membrane disruption does not appear to be the main function of either cementoin, elafin or full-length pre-elafin/trappin-2 against *P. aeruginosa*. Our results rather suggest that pre-elafin/trappin-2 and elafin, but not cementoin, possess the ability to modulate the expression of some *P.aeruginosa *virulence factors, possibly through acting on intracellular targets.

## Background

*Pseudomonas aeruginosa *is a Gram-negative bacterium that rarely causes serious infections in healthy individuals. It is, however, the prevalent opportunist pathogen encountered in nosocomial infections and the major etiologic agent responsible for the morbidity, clinical deterioration and early mortality associated with patients suffering from cystic fibrosis (CF) [1-5]. A plethora of virulence factors expressed by *P. aeruginosa *is associated with acute and chronic infections [6]. Perhaps the most dramatic change that characterizes *P. aeruginosa *chronic infections is the transformation from a non-mucoid to a mucoid phenotype [7]. This is associated with an overproduction of alginate, which favors biofilm formation and an increased antibiotic resistance [8]. Chronic pseudomonal infections are thought to be virtually impossible to eradicate and the current strategy in the management of CF patients, which become infected in their early childhood, is to prevent or retard progression to chronic infection by treating *P. aeruginosa *infections with conventional antibiotic therapy as soon as they appear [9,10].

In this era of increased antibiotic resistance, the development of novel antimicrobial agents is urgently needed. In the past decade, gene-encoded short positively charged peptides, collectively known as antimicrobial peptides (AMP), have attracted much attention because of their broad antimicrobial activities and their potential use as therapeutics [11-18]. AMP are characterized by their short length (12-50 aa), polycationic (at least +2 net charge as Lys or Arg) and, usually, amphipathic characters. Among the nearly thousand identified peptides from various organisms, four classes can be distinguished based on their structures; (i) amphipathic α-helical, (ii) β-sheet structures; (iii) extended structures, and (iv) hairpin loop stabilized by a single disulfide bridge. A common feature ascribed to AMP is their ability to interact with the negatively charged bacterial membranes and polyanionic cell surface (lipopolysaccharide (LPS) of Gram-negative and lipoteichoic acid of Gram-positive bacteria). At their lethal concentrations *in vitro*, they generally disrupt membrane integrity and cause bacterial lysis. Some AMP, however, do not cause membrane disruption, but act on intracellular targets such as nucleic acids [19].

We are studying the human multifunctional innate defense molecule known as pre-elafin/trappin-2. This protein is composed of two domains, an N-terminal moiety of 38 aa known as cementoin based on its ability to be cross-linked to extracellular matrix proteins through the action of a transglutaminase and a C-terminal part of 57 aa, or elafin domain, that displays sequence similarity with whey acidic protein (WAP) [20]. This latter domain is a potent and specific inhibitor of neutrophil elastase (NE) and myeloblastin, as well as pancreatic elastase [21,22]. Its structure was determined both by X-ray crystallography in complex with pancreatic elastase and free in solution by nuclear magnetic resonance (NMR) spectroscopy [23,24]. The salient structural feature of elafin is a β-sheet stabilized by three disulfide bridges along with an inhibitory loop connected to the central β-sheet by a fourth disulfide bridge. There is no structural information regarding the cementoin domain or the full-length pre-elafin molecule.

Apart from the well-known inhibitory and anti-inflammatory properties of pre-elafin/trappin-2, previous studies also established that the full-length molecule and each of its domains possess broad antimicrobial activity, namely against the bacteria *P. aeruginosa *and *S. aureus*, and the yeast *C. albicans *[25-28]. Furthermore, adenoviral overexpression of pre-elafin/trappin-2 in a mouse model of acute *P.aeruginosa *infection was shown to reduce the bacterial load and to facilitate clearance of the microorganism [29]. Although it has been documented that the full-length molecule is more active than its constituent domains *in vitro *[25,27,28], the exact mechanism of action of each of these peptides against microbial infections is largely unknown. We recently reported that the variable sensitivity of *P. aeruginosa *strains to pre-elafin/trappin-2 could be partly explained by the specific inhibition of a peptidase secreted by some, but not all, strains by the elafin domain [27]. However, both domains also display antimicrobial activity independent from the peptidase inhibitory function of elafin suggesting that the antimicrobial properties of these peptides are the sum of several unique attributes [27,28].

In the present study we have determined the secondary structures of the cementoin peptide in the presence or absence of membrane mimetics. This peptide is essentially unstructured in aqueous solution but, like a large class of AMPs, adopts an α-helical conformation in the hydrophobic membrane environment. However, when compared with magainin-2, a typical α-helical AMP with potent lytic activity [30], the lytic properties of cementoin, elafin or pre-elafin/trappin-2 toward *P. aeruginosa *and artificial membranes are very weak. We have also tested the ability of pre-elafin/trappin-2 and its domains to interfere with the expression of known *P. aeruginosa *virulence factors and compared this activity to that of azithromycin, an antibiotic that perturbs cell to cell communication in *P. aeruginosa *and significantly retards biofilm formation [31,32]. Pre-elafin/trappin-2 and elafin, but not cementoin, were found to reduce biofilm development and the secretion of pyoverdine and this correlated with the ability of these peptides to bind DNA *in vitro *and to accumulate within the bacterial cytosol. Rather than causing extensive cell lysis, our data thus suggest that pre-elafin/trappin-2 and elafin attenuate the expression of some *P. aeruginosa *virulence factors, possibly through acting on an intracellular target.

## Results

### The cementoin domain of pre-elafin/trappin-2 adopts an α-helical conformation in the presence of membrane mimetics

**Figure 1:**
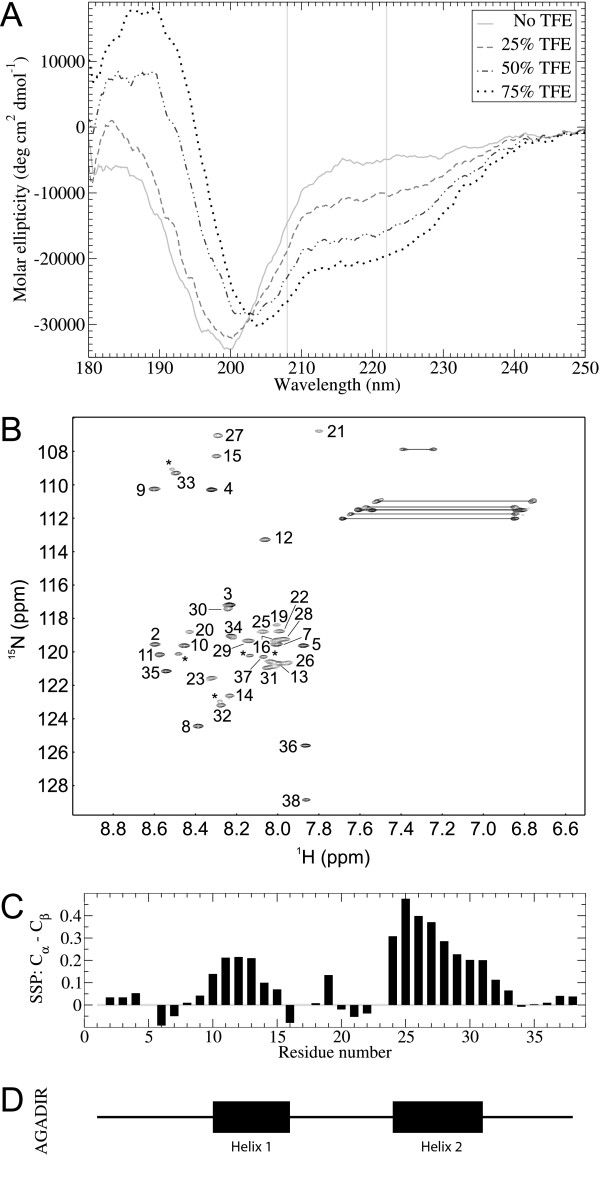
**Biophysical characterization of cementoin**. A) CD spectra of cementoin with varying concentrations of TFE (up to 75%). The vertical lines indicate 208 and 222 nm, *i.e*. characteristic wavelengths for assessing the presence of α-helices. B) 2 D ^15^N-HSQC spectrum of cementoin in the presence of 50% TFE. Backbone assignments are shown. Side-chain Asn, Gln and Arg doublets are depicted with a line between the two resonances while unassigned additional peaks (potentially arising from slow exchange, see text) are labeled by an asterisk (*). C) SSP analysis of backbone Cα and Cβ chemical shifts. Secondary structure predictions depend on the result where positive values infer α-helices and negative values, β-sheets. D) Secondary structure predictions from AGADIR with α-helices shown as black boxes.

Different experiments were performed to characterize the structure of cementoin and its interaction with membranes. First, we recorded circular dichroism (CD) spectra in the presence or absence of trifluoroethanol (TFE), which mimics a membrane environment [33] (Fig. 1A). In an aqueous solution, the CD spectrum is typical of an unstructured protein with a prominent negative peak at 199 nm. When TFE was added, the intensity of this peak decreased concomitantly with the appearance of minima around 205 nm and 222 nm whose intensity increased with the concentration of TFE. This is characteristic of an α-helical structure and the α-helical content of cementoin was estimated to be 48% in 50% TFE and up to 58% in 75% TFE. The observed isodichroic point at 203 nm indicates that the transition between the unstructured to the α-helical conformation is a two-state transition. Hence, a hydrophobic environment either induces or stabilizes α-helices in cementoin. This is in agreement with the AGADIR algorithm (Fig. 1D), which predicts the formation of two α-helices in cementoin: helix 1 with residues 10- 16 and helix 2 with residues 24-31, for a predicted total α-helical content of 39%.

Using NMR, such a formation of structure upon addition of TFE was also apparent from the more dispersed ^1^H chemical shifts observed in the presence of 50% TFE (data not shown). These conditions were thus chosen to determine the secondary structures of cementoin. A series of triple-resonance spectra were recorded in order to assign backbone chemical shifts (Fig. 1B). From the assigned backbone chemical shifts, it was possible to predict secondary structures using the SSP approach (*see Methods*). This yielded two predicted helices in cementoin (Fig. 1C), similar to that predicted by AGADIR (Fig. 1D). Atomic resolution on spin relaxation data (R_1_, R_2_, NOE; see additional file 1: Fig. S1 A) confirmed most of AGADIR predictions. Indeed, residues for which high flexibility is inferred (from reduced spectral density mapping of spin relaxation data, see Fig. S1 B & C) are those located right before helix 1 as proposed by AGADIR, and directly after helix 2. Additionally, R_2 _data with higher values within proposed α - helices, but also in the middle of the peptide would tend to indicate that this whole section of the peptide is in slow exchange. Hence, both proposed α-helices could be nucleating points where α - helical structures would start appearing, enabling the transient existence of a long α-helix spanning residues 10-31. Of course, this structure would be transient as the NOE values are quite low (~0.5) for this whole stretch.

We previously showed that pre-elafin/trappin-2, elafin and particularly the cementoin domain interact strongly with negatively charged liposomes composed of phosphatidyl glycerol (PG) [27]. We used NMR with bicelles composed of a mixture of dihexanoyl phosphatidylcholine (DHPC), dimyristoyl phosphatidylcholine (DMPC) and dimyristoyl phosphatidylglycerol (DMPG) to a final ratio of 8:3:1 to characterize this interaction, by measuring the translational diffusion coefficients for cementoin in the absence and presence of bicelles (Table 1 and additional file 1: Fig. S2). In the presence of bicelles, cementoin diffused with a rate much slower (1.24 × 10^-6 ^cm^2^.s^-1^) than in an aqueous environment (4.28 × 10^-6 ^cm^2^.s^-1^). It is important to note here that this effect of bicelles on slowing the diffusion of cementoin is not caused by an increase in solvent viscosity, since water was found to diffuse at approximately the same rate in both conditions (Table 1). This slower rate is close to that measured for the bicelles alone (0.79 × 10^-6 ^cm^2^.s^-1^; Table 1 and Fig. S2). This finding convincingly demonstrates that an interaction exists between cementoin and bicelles. From these data, the fraction of cementoin bound to bicelles was estimated to be 87% (see *Methods*), implying that ~13% cementoin would be free in solution. These numbers could also mean that the interaction is weak and transient and that, at a given moment, 87% of cementoin are bound. The exact mechanism of interaction with membranes would depend on whether the α-helical structures in cementoin are limited to those two α -helices proposed by AGADIR and chemical shifts or to a longer α -helix spanning residues 10-31 that would allow penetration of cementoin through the entire membrane width. Our diffusion data cannot discriminate between these different possibilities.

**Table 1:** Diffusion behavior of cementoin in H_2_O and bicelles.

Experimental condition	H_2_O	DHPC	DMPC^1^	cementoin (amide)^2^	cementoin (aliphatic)^3^
cementoin	25.22	-	-	4.27	4.28

DHPC: DMPC: DMPG (8:3:1)	21.07	0.68	0.38	-	-

DHPC: DMPC: DMPG (8:3:1) + cementoin	21.08	0.97	0.61	1.25	1.23

Diffusion coefficients* are displayed for bicelles (DHPC + DMPC), H_2_O and cementoin in either of three experimental conditions in units of 10^-6 ^cm^2^/s.

* Calculated from A_G _= A_0 _exp[-(γδG)^2 ^(Δ - δ/3) D_s _]

^1 ^DMPG resonance was not observed and assumed to be overlapped with DMPC.

^2 ^From an isolated resonance at 7.4 ppm.

^3 ^Values are the average of three different resonances at 2.0, 2.1 and 3.0 ppm.

### Binding of pre-elafin/trappin-2 peptides to *P. aeruginosa *or artificial membranes does not cause extensive membrane disruption

**Figure 2:**
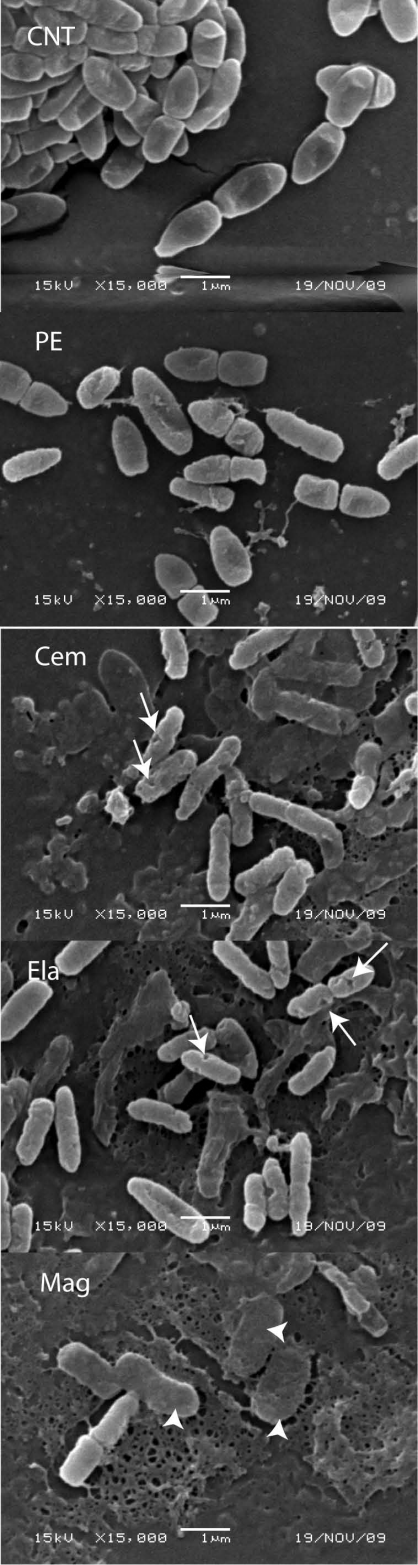
**Scanning electron micrographs of *P. aeruginosa *incubated with cementoin, elafin, pre-elafin/trappin-2 or magainin 2**. *P. aeruginosa *(~1 × 10^7 ^in 500 μL) were incubated 2 h with the indicated peptides before being processed for scanning electron microscopy as described in *Methods*. CNT; control performed in the absence of peptides, PE; pre-elafin/trappin-2, Cem; cementoin, Ela; elafin, Mag; magainin 2. White arrows point to pore-like structures and white arrowheads to ghost bacterial cells.

Positively charged α-helical peptides like cementoin, are characteristic of many AMPs. These were previously shown to either disrupt membranes and cause bacterial lysis or to translocate into the bacterial cytoplasm without causing cell lysis [19]. To obtain information about the mode of action of recombinant cementoin compared with that of elafin and pre-elafin/trappin-2 on *P. aeruginosa*, we first examined the effect of these peptides on bacteria by scanning electron micrography (SEM). As shown in Fig. 2, both elafin and cementoin significantly modified the appearance of *P. aeruginosa *cell surface with clear evidence of wrinkling, blister formation and the presence of pore-like structures (white arrows in Fig. 2). At the same concentration, pre-elafin/trappin-2 appeared to affect less severely the bacterial morphology and cells harboring pore-like structures were much less abundant. The presence of pores suggests that membrane integrity is compromised by addition of these peptides. However, ghost cells were rarely observed. In sharp contrast, when *P. aeruginosa *were exposed to magainin 2, a lytic AMP, much fewer cells could be visualized by SEM and ghost cells were numerous indicating cell lysis (white arrowheads in Fig. 2).

**Figure 3:**
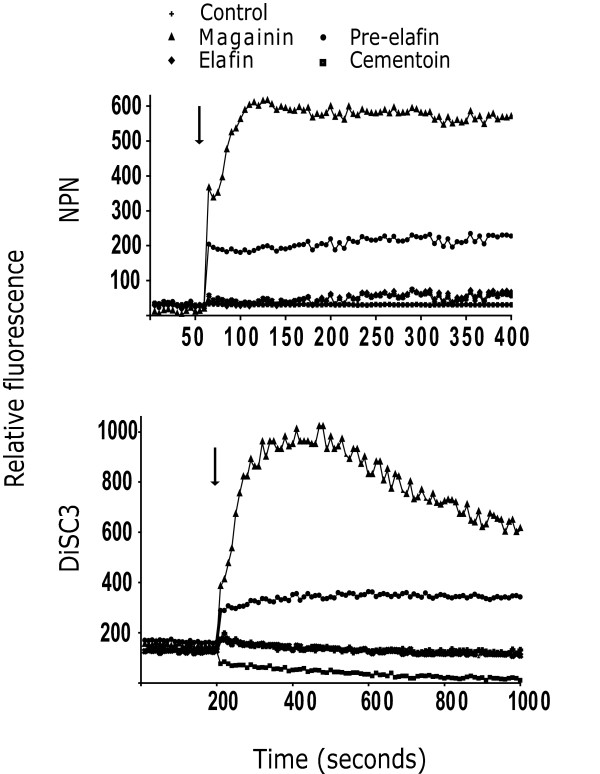
**Depolarization of *P. aeruginosa *membranes upon incubation with magainin 2, pre-elafin/trappin-2 or derived peptides**. Fluorescence emission (arbitrary units) of the probe NPN inserted into the outer membrane (*top panel*) or the probe DiSC_3 _inserted into the inner membrane (*bottom panel*) of *P. aeruginosa *upon addition of the indicated peptides. The controls were performed in phosphate buffer alone. Pre-elafin/trappin-2 and elafin were used at 20 μM, cementoin at 100 μM and magainin 2 at 10 μM. The arrow indicates the time-point for the addition of the various peptides.

To further document that membrane disruption may not be the primary role of cementoin, elafin and pre-elafin/trappin-2, the ability of these peptides to cause membrane depolarization using the fluorescent probes, 1-N-phenylnaphthylamine (NPN) and 3,3'- dipropylthiacarbocyanine (DiSC_3_) was tested. NPN is a neutral hydrophobic probe that is excluded by an intact outer membrane, but is taken up into the membrane interior of an outer membrane that is disrupted by antimicrobial peptide action [34]. NPN fluoresces weakly in free solution but strongly when it crosses the outer membrane barrier into the cell. As shown in Fig. 3 (*top panel*), upon addition of 10 μM magainin 2 a sharp increase in fluorescence was observed. The addition of 20 μM pre-elafin/trappin-2 led to a much weaker fluorescence signal, and 100 μM cementoin or 20 μM elafin had no effects on membrane depolarization. No variation of fluorescence was seen upon addition of NPN to bacterial cells when no peptide was added. To evaluate the effects of the recombinant peptides on *P. aeruginosa *cytoplasmic membrane, the fluorescent probe DiSC_3 _was used. DiSC_3 _distributes between the cells and the medium. This cationic dye concentrates in the cytoplasmic membrane under the influence of the membrane potential resulting in a self-quenching of fluorescence. If the membrane is depolarized, the probe will be released into the medium, causing a measurable increase in fluorescence [35]. The assays were again compared with magainin 2, which can permeabilize the bacterial membranes. In contrast to a strong release of fluorescence upon addition of magainin 2, pre-elafin/trappin-2 and derived peptides weakly, if at all, induced fluorescence emission (Fig. 3; *bottom panel*). Our results suggest that pre-elafin/trappin-2 and derived peptides, in contrast to magainin 2, acted on the outer and inner membranes without causing extensive membrane depolarization.

We also addressed the lytic properties of these peptides by measuring the release of calcein entrapped within PG-composed liposomes. A 15-min exposure of liposome-entrapped calcein with magainin 2 led to a 32% release of calcein relative to that measured for liposomes permeabilized with 1% Triton X-100. In contrast, no more than 5% of calcein was released by either cementoin, elafin or pre-elafin/trappin-2. We thus conclude that these peptides have very weak lytic activities. This explains our finding that no measurable MIC (minimal inhibitory concentration) could be measured even if high concentrations of peptides were tested (up to 128 μg/mL for pre-elafin/trappin-2 and elafin and up to 256 μg/mL for cementoin).

### Fluorescein-labeled pre-elafin/trappin-2 incubated with *P. aeruginosa *accumulates within the cytosol and both elafin and pre-elafin/trappin-2 bind DNA *in vitro*

**Figure 4:**
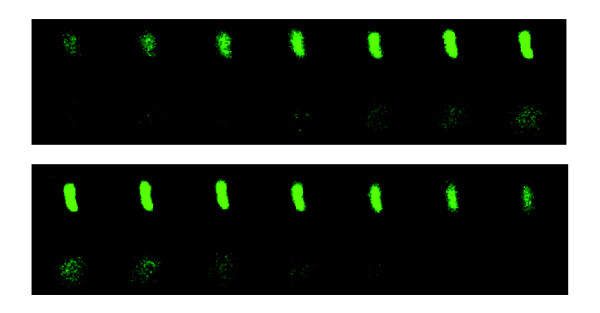
**Confocal microscopy of *P. aeruginosa *incubated with fluorescein-labeled pre-elafin/trappin-2**. Mid-logarithmic phase cultures of *P. aeruginosa *were incubated for 1 h at 37°C with fluorescein-labeled pre-elafin/trappin-2 and observed by confocal microscopy at 400 × magnification. From left to right, consecutive 0.2 μm sections of a fluorescent bacterial cell.

Weak membrane depolarization and leakage of liposome-entrapped calcein, while indicating little membrane disruption, does not exclude that transient pores may form upon incubation of *P. aeruginosa *with pre-elafin/trappin-2 and derived peptides, as suggested by SEM examination. Formation of transient pores could lead to the translocation of the peptides across membranes. We previously reported that fluorescein-labeled pre-elafin/trappin-2 heavily decorated *P. aeruginosa *cells as assessed by fluorescence microscopy [27]. Here we used confocal microscopy to examine the fate of fluorescein-labeled pre-elafin/trappin-2 upon a 1 h incubation with *P. aeruginosa*. As shown in Fig. 4, the whole bacterial cell was fluorescent in all consecutive 0.2 μm sections. This is taken as evidence that pre-elafin/trappin-2 not only binds the surface, but also accumulates within the bacterial cytosol.

**Figure 5:**
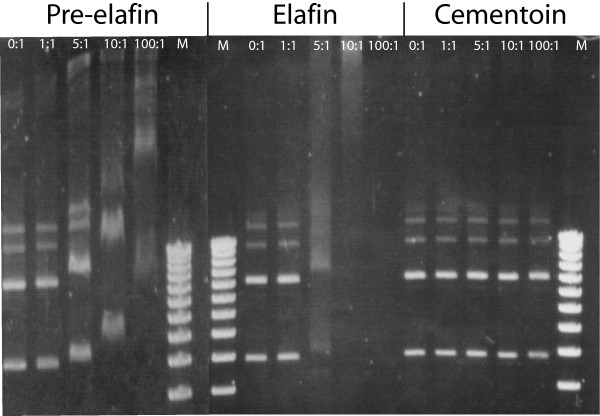
**Electrophoretic mobility shift assay of plasmid DNA incubated in the absence or presence of pre-elafin/trappin-2, elafin and cementoin**. Plasmid pRS426 (100 ng) was incubated with the indicated ratios of peptide/DNA (w/w) for 1 h and then analyzed by agarose gel electrophoresis followed by staining with ethidium bromide. Above are representative gels from an experiment performed in triplicata. M; 1 kb DNA ladder from Invitrogen.

Given the polycationic character of pre-elafin/trappin-2 and derived peptides and the apparent ability of pre-elafin/trappin-2 to traverse lipid bilayers, we considered the possibility that they could interact with nucleic acids. To test this hypothesis, we evaluated whether any of the pre-elafin/trappin-2 and derived peptides could induce an electrophoretic mobility shift (EMSA) of DNA. As shown in Fig. 5, the EMSA assay revealed that pre-elafin/trappin-2 binds to DNA *in vitro *at a peptide:DNA ratio of 5:1 and greater. Similar results were also obtained with the elafin domain. In contrast, no DNA shift was observed for the cementoin peptide up to a 100:1 ratio. Hence, despite the fact that the cementoin peptide has a greater positive charge (+4) than elafin (+3), the structure of the elafin domain appears necessary and sufficient for binding to DNA *in vitro*.

### Pre-elafin/trappin-2 and elafin attenuate the expression of known *P. aeruginosa *virulence factors

To test whether the binding and/or translocation of the pre-elafin/trappin-2 and derived peptides could modify the behavior of *P. aeruginosa*, we assayed the expression of known virulence factors in the absence or presence of the various peptides and this was compared to that observed in the presence of azithromycin. At sublethal concentrations, azithromycin is known to interfere with the quorum sensing of *P. aeruginosa *and this was reported to reduce the expression of numerous genes encoding virulence factors as well as to retard formation of a biofilm [31,32,36]. We specifically assayed for the secretion of the siderophore pyoverdine, the peptidase lasB, the production of alginate and the development of a biofilm. Apart from the biofim development, which was estimated after 26 h of growth in the presence or absence of peptides, all assays were carried out on 24 h cultures of *P. aeruginosa*. As shown in Table 2, pre-elafin/trappin-2 was the most effective peptide in all assays, and at 8 μM it reduced the secretion of pyoverdine and the formation of a biofilm by ~40%. At this concentration, it also reduced by approximately 25% the secretion of lasB and alginate although not in strictly dose-dependent manner. Interestingly, the effect of pre-elafin/trappin-2 paralleled that of azithromycin used at the same concentrations. Compared to pre-elafin/trappin-2 and azithromycin, the elafin peptide was only modestly less efficient with an observed ~30% reduction on the secretion of pyoverdine and biofilm formation. The cementoin peptide alone barely (4 μM) or modestly (8 μM) affected the expression of these virulence factors. Hence, both pre-elafin/trappin-2 and elafin appear to attenuate the expression of some *P. aeruginosa *virulence factors and this correlates with their ability to bind DNA *in vitro*.

**Table 2:** Attenuation of *P. aeruginosa *virulence factors by pre-elafin/trappin-2, elafin and cementoin

Peptide	[μM]	%^1 ^Pyoverdine	% *Las B*	% Alginate	% Biofilm
Pre-elafin/trappin-2	4	71 ± 2	83 ± 2	76 ± 2	70 ± 2
	8	59 ± 2	75 ± 2	72 ± 2	57 ± 4

Elafin	4	82 ± 2	87 ± 4	79 ± 3	86 ± 2
	8	69 ± 1	73 ± 5	77 ± 2	69 ± 2

Cementoin	4	96 ± 2	96 ± 4	95 ± 1	94 ± 2
	8	91 ± 1	88 ± 4	87 ± 2	87 ± 2

Azithromycin	4	69 ± 2	85 ± 4	80 ± 3	62 ± 4
	8	55 ± 2	76 ± 2	75 ± 3	44 ± 5

^1^The results are expressed as a percentage ± SD relative to *P. aeruginosa *cultures grown in the absence of peptides, which were set at 100%. For the assays of pyoverdine and lasB the values represent the mean of 3 experiments performed in duplicata. For the assays of alginate and biofilm formation the values represent the mean of 3 experiments.

## Discussion

The aim of the present study was to determine the secondary structures of the N-terminal moiety of pre-elafin/trappin-2 (cementoin) and to investigate the mode of action of this peptide compared to elafin and pre-elafin/trappin-2 against *P. aeruginosa*. We have shown here by CD and NMR studies that the cementoin peptide is essentially unstructured in aqueous solution but adopts an α-helical conformation in the presence of a membrane mimetic (TFE). This characteristic is shared with the most important class of AMP, the linear polycationic peptides [33], which include the human LL-37 peptide [37]. Whilst TFE is known to induce α-helical structures by favoring intra hydrogen bonding, it has been demonstrated for a large number of AMP that this propensity to adopt an α-helical conformation in TFE is also observed in the presence of artificial membranes that more closely mimic the physiological environment [33]. Hence, the secondary structures determined for cementoin in the presence of TFE are likely to be physiologically relevant. Previous studies showed that cementoin binds to the lipid core of lipopolysachharide (LPS) [27,38] as well as to artificial membranes, particularly the negatively charged membranes enriched in PG [27]. We confirmed here these finding by demonstrating that the translational diffusion of cementoin in the presence of DMPG-containing bicelles is considerably slower than that of free cementoin. Furthermore, we estimated that under the conditions used (peptide:lipid millimolar ratio of 1:200), approximately 87% of the cementoin peptide was bound to bicelles.

As revealed by SEM, binding of cementoin to *P. aeruginosa *elicited obvious morphological changes such as wrinkling and blister formation on the cell surface and the presence of pore-like structures. This is reminiscent to that described earlier for the binding of pre-elafin/trappin-2 to *P. aeruginosa *by Baranger *et al. *[28]. However, in our hands the morphological changes induced by pre-elafin/trappin-2 were not as severe as those reported earlier or to that observed in the present study with cementoin and elafin alone. The reason for this apparent discrepancy is not clear but could be due to a different peptide to bacteria ratio and/or to the actual fraction of mature elafin present in the two preparations of pre-elafin/trappin-2. It is generally assumed that the presence of pore-like structures is indicative of cell lysis. However, several lines of evidence suggest that the membrane disruption properties of cementoin, elafin and pre-elafin/trappin-2 are considerably weaker compared to that of the amphibian lytic AMP magainin 2. First, unlike that observed with pre-elafin and derived peptides, numerous ghost cells were visualized by SEM upon incubation of *P. aeruginosa *with magainin 2. Second, compared to this AMP, outer and inner membrane depolarization by pre-elafin/trappin-2, elafin and cementoin, as measured with the probes NPN and DiSC_3_, were significantly weaker. Third, the release of liposome-entrapped calcein by magainin 2 was six-fold greater than that measured with any of the pre-elafin/trappin-2 derived peptides. Finally, no MIC values could be determined for cementoin, elafin or pre-elafin/trappin-2 compared to a MIC of 8 μg/mL for magainin 2 against *P. aeruginosa *ATCC 27853 strain [39]. We therefore tentatively conclude that membrane disruption *per se *may not be the main function of these peptides *in vivo*.

Historically, the lytic properties of a peptide were important criteria to classify it as an AMP. It is however becoming increasingly documented that several AMP possess other functions such as modulating the host response, through interacting with innate defense molecules, or modifying the microbial behavior by acting on intracellular targets [19,40,41]. In line with this notion, pre-elafin/trappin-2 was recently proposed to opsonize *P. aeruginosa *to facilitate its clearance by macrophage [42]. In the present work, we provided evidence that pre-elafin/trappin-2 may also traverse membranes, presumably to act on intracellular targets. A potential target could be DNA as both elafin and pre-elafin/trappin-2 were shown to bind DNA *in vitro *and this correlated with their ability to attenuate the expression of some *P. aeruginosa *virulence factors (*see below*). Buforin II is perhaps the best-documented AMP that acts on an intracellular target, the nucleic acids [43,44]. Investigation of the membrane translocation mechanism of buforin II led to the proposal that this peptide induces the formation of a toroidal pore similar to that described for magainin 2 [45]. However, unlike magainin 2, the short lifetime of the pore enables translocation of the peptide without causing membrane permeabilization and leakage of the intracellular content. The weak membrane depolarization and calcein release observed with pre-elafin/trappin-2 and elafin suggest that these peptides might be similarly translocated across lipid bilayers without causing extensive cell lysis. However, we cannot exclude the possibility that like Gramicidin A the size of the pores, rather than their lifetime, explains the weak membrane depolarization and calcein release observed [46]. Future investigations using solid-state NMR to further characterize the interaction between pre-elafin/trappin-2 peptides and model membranes are needed to confirm their translocation properties and the exact mechanism involved.

Azithromycin is not considered an effective antibiotic against *P. aeruginosa *due to its high MIC value (> 64 μg/mL; [31,47]). Yet, at sublethal concentrations for *P. aeruginosa*, azithromycin was found to retard biofilm formation [32] and to reduce the production of alginate, pyocyanin and the secretion of elastase (lasB) [31,36]. We confirmed here these previous data and showed that it also reduces secretion of the siderophore pyoverdine. Both pre-elafin/trappin-2 and elafin were found to similarly affect the expression of *P. aeruginosa *virulence factors, namely the biofilm formation and the secretion of pyoverdine. Because these peptides were previously found to reduce the plating efficiency (cfu) of *P. aeruginosa *following a 3 h incubation with the peptides in phosphate buffer [25,27], it could be argued that the observed attenuation on the expression of virulence factors is indirect. However, we believe this is unlikely for three reasons. First, all phenotypes were tested following prolonged incubation periods (ranging from 24 to 26 h) with the peptides in PSB medium. Under these conditions, the A_595 nm _of the cultures at the end of the incubation were almost undistinguishable between samples incubated in the presence or absence of peptides. Second, all phenotypes were quantified taking into account the final A_595 nm _of the cultures. Finally, whereas the plating efficiency of *P. aeruginosa *following a 3 h incubation with the peptides in phosphate buffer varied considerably between different strains (*i.e*. ATCC 27853 vs ATCC 33348; [25,27]), this was not found to be the case for the reduced biofilm formation and secretion of pyoverdine between these two strains (data not shown). In further support to the role of pre-elafin/trappin-2 in the attenuation of *P. aeruginosa *virulence factors, it was recently reported that the A549 cell line expressing pre-elafin/trappin-2 reduces both the number of bacteria and the area of growing *P. aeruginosa *biofilm by approximately 50% [48]. Although the effect of pre-elafin/trappin-2 and elafin is modest *in vitro*, this may contribute *in vivo*, along with the anti-inflammatory properties of these molecules, to prevent against *P. aeruginosa *infections.

## Conclusions

We have demonstrated that the N-terminal moiety of pre-elafin/trappin-2 (cementoin) adopts an α-helical conformation in the presence of a membrane mimetic, which is typical of a large class of AMP. Despite the morphological changes observed at the surface of *P. aeruginosa *in the presence of cementoin, elafin or pre-elafin/trappin-2, the membrane disruption properties of these peptides are weak compared to magainin 2. We provided evidence that pre-elafin/trappin-2 and elafin may act on an intracellular target, possibly DNA. Although future studies on the interaction of these peptides with artificial membranes are needed to confirm and to elucidate the mechanism of membrane translocation, both pre-elafin/trappin-2 and elafin were shown to attenuate the expression of some *P. aeruginosa *virulence factors, which may contribute to the defense against *P. aeruginosa *infection.

## Methods

### Bacterial, yeast strains and growth conditions

*P. aeruginosa *strain ATCC #33348 was used in all functional assays with the pre-elafin/trappin- 2 and derived peptides. Bacteria were grown at 37°C with (250 rpm) or without agitation in peptone soy broth (PSB). *E. coli *strain BL21(DE3) (Novagen, Mississauga, ON, Canada) was used for the recombinant production of the cementoin peptide. The *S. cerevisiae *yeast strain YGAU-Ela2 (Matα *his3 leu2 ura3 mf*α*1*/*mf*α*2Δ::LEU2 yps1Δ::HIS3 ura3::pGAU-Ela2*) was used for the production of pre-elafin/trappin-2. This strain was constructed by integrating the plasmid pGAU-Ela2 (*see below*) digested by *Nco*I at the *ura3 *locus of the YBAD1 strain previously used for the production of pre-elafin/trappin-2 [49]. Yeast cells were grown at 30°C in yeast dextrose peptone (YPD) medium.

### Plasmids, oligonucleotides and DNA manipulations

DNA manipulations, bacterial and yeast transformations were all carried out according to standard procedures [50,51]. Unless otherwise indicated, all restriction and DNA-modifying enzymes were purchased from New England Biolabs Ltd (Pickering, ON, Canada). The bacterial expression plasmid pET32-cem has been described previously for the production of the cementoin domain [27]. The yeast integration plasmid pGAU-Ela2 was constructed by first excising the 2 μ origin of pVT-Ela2 through digestion with *Bst*X1 and *Sma*I, fill-in with the Klenow fragment and ligation. Next, the *GAL1 *promoter obtained as an *Eco*RI-*Bam*HI fragment from plasmid pJK6 [52] was blunt-ended with Klenow and inserted into the unique *Pvu*II site located upstream of the pre-elafin fusion protein in pVT-Ela2 [49]. The resulting integration plasmid was named pGAU-Ela2. All DNA constructs were verified for integrity by DNA sequencing.

### Production and purification of recombinant pre-elafin and cementoin

Growth conditions for the production of bacterially expressed cementoin peptide were as described previously [27]. For the production of pre-elafin/trappin-2, the yeast YGAU-Ela2 strain was first cultured 2 days at 30°C in 3 L of YPD with daily adjustments of the pH (pH 6.0) and addition of dextrose (1% w/v). The culture medium was then replaced by 1 L of synthetic complete -uracil medium supplemented with galactose 2% and the culture was resumed for another 2 days at 30°C with twice daily adjustments of the pH and additions of yeast nitrogen base (1% w/v) and galactose (1% w/v). Uniformly ^15^N-^13^C-labeled cementoin samples for NMR spectroscopy were prepared using ^15^NH_4_Cl and [^13^C]-glucose (Cambridge Isotope Laboratories, Andover, MA) as the sole nitrogen and carbon sources, as previously described [53]. Induction with 1 mM isopropyl-β-D-thiogalactopyranoside (IPTG) was performed for 16 h at 37°C.

Purification of recombinant His-tagged pre-elafin/trappin-2 from yeast culture supernatants was essentially as described [49,54], except the diafiltration proceeded in two steps. The permeate from a first diafiltration performed with the cleared supernatant over a 30-kDa cartridge was followed by concentration on a 3-kDa cartridge. Purification of the cementoin peptide from bacterial pellets, either uniformly labeled or not, was as previously described [27]. Purified peptides were concentrated in deionized water using stirred-cells, lyophilized and stored at -80°C until use. Recombinant human elafin was purchased from AnaSpec (San Jose, CA, USA).

### Structural analysis

CD spectra were recorded using a JASCO J-710 instrument upgraded to J-715 by varying wavelengths between 180 and 250 nm with steps of 0.2 nm. Cementoin was prepared at a concentration of 1 mg/ml in water supplemented with 0% to 75% TFE. The alpha helical content was obtained from the following formula:

$$\text{H}\alpha =100*(\theta_{222\text{nm}}-3000)/-39000,$$

where Hα is the percent α-helix and θ, the molar ellipticity per residue (deg.cm^2^.dmol^-1^), was defined as follows:

θ=ΔA.MW/C.n.l,

where MW is the peptide molecular weight (here 3948.54 g/mol), n is the number of residues in the peptide (here 38 residues), C is the peptide concentration (here 1g/L), and l is the length of the optical course (here 0.01 cm).

The AGADIR software http://agadir.crg.es/ developed by the Serrano's group [55-59] was used to predict the cementoin secondary structures. The parameters for ionic strength, temperature and pH were set to 1 M, 278°K and 7.0, respectively.

NMR samples were prepared by dissolving lyophilized protein in an aqueous solution at pH 6.4 to a final concentration of 0.5 mM and with 60 μM 2,2-dimethylsilapentane-5-sufonic acid and 10% D_2_O (for chemical shift referencing and locking, respectively). The spectra were recorded at a temperature of 2°C (calibrated with MeOH) on a 600 MHz Varian INOVA spectrometer equipped with either a room temperature triple resonance probe or a z-axis pulsed-field gradient triple resonance cold probe. Two-dimensional ^15^N-HSQC, 3D-HNCO, 3D-HN(CO)CA, and 3D-CBCA(CO)NH spectra (Biopack, Varian Inc., Palo Alto, CA) were recorded. NMR data were processed with NMRPipe/NMRDraw [60] and analyzed with NMRView [61]. Backbone assignments proceeded within Smartnotebook v5.1.3 [62]. The chemical shift index was calculated for both C_α _and C_β _for secondary structure prediction using the SSP approach [63].

Experiments for the measurement of diffusion coefficients by NMR were performed for cementoin in the absence and presence of bicelles. The procedure used was as described previously [64]. In summary, the bicelles used were a mixture of DHPC, DMPC and DMPG for a final ratio of 8:3:1 (with a (DMPC+DMPG)/DHPC ratio, *i.e*. long-chain to short-chain or q ratio, of 0.5). Experiments were performed with cementoin at 0.5 mM and were recorded at 37°C. Rates were extracted using the following equation:

$$A_{G}=A_{0}\exp[-{\left(\gamma \delta G\right)}^{2}(\Delta -\delta /3)D_{s}],$$

Where γ is ^1^H gyromagnetic ratio (2.6753 × 10^4 ^rad.s^-1^.G^-1^), δ is the duration of the pulse -field gradient (PFG, 0.4 s), G is the gradient strength (from 0.5 to 52 G.cm^-1^), Δ is the time between PFG trains (0.154 s) and D_s _is the diffusion coefficient (in cm^2^.s^-1^).

The fraction of cementoin bound to bicelles was estimated with the following equation:

$${\text{D}}_{\text{obs}}={\text{p}}_{\text{free}}{\text{.D}}_{\text{free}}+{\text{p}}_{\text{bound}}{\text{.D}}_{\text{bound}},$$

where D_obs_, D_free _and D_bound _are the diffusion coefficients for all cementoin states (observed rate: 1.24 cm^2^.s^-1^), for free cementoin (4.28 cm^2^.s^-1^) and for bound cementoin (by approximation, for bicelles: 0.79 cm^2^.s^-1^), respectively, and p_free _and p_bound _are the fractions for free and bound cementoin (with p_free _+ p_bound _= 1), respectively.

Backbone chemical shifts and spin relaxation data were deposited in the BMRB under accession number 16845.

### Scanning electron micrography

Scanning electron micrography (SEM) of *P. aeruginosa *(~1 × 10^7 ^in 500 μL) incubated for 2 h in the absence or presence of the indicated peptides (8 μM) was performed on a JEOL JSM-6360LV microscope essentially as described [28], except samples were placed on Alcar© films and dehydrated by a series of incubation in alcohol (50, 70, 95 and 100%).

### Outer and inner membrane depolarization of *P. aeruginosa*

The outer membrane depolarization activity of the recombinant peptides was determined by the 1-N-phenylnaphthylamine (NPN) uptake assay of Loh *et al. *[34] with intact cells of *P. aeruginosa *using the Fluorescan Ascent FL microplate fluorometer. *P. aeruginosa *was grown with agitation to an A_600 nm _= 0.6 and harvested by centrifugation. The cells were washed in 5 mM HEPES, pH 7.8 and resuspended to an A_600 nm _of 0.5 in the same buffer. The microtiter plate wells were supplemented with cells (200 μL) and NPN dissolved in acetone was added to a final concentration of 10 μM. Then peptides were added to the desired concentration and the intensity of fluorescence was measured at λ_ex _= 355 nm and λ_em _= 444 nm.

The cytoplasmic membrane depolarization activity of the peptides was determined as previously described with the membrane potential-sensitive dye DiSC_3 _[35]. Briefly, *P. aeruginosa *was grown at 37°C with agitation to an A_600 nm _of 0.6 and harvested by centrifugation. The cells were washed in 5 mM HEPES, pH 7.8 and resuspended to an A_600 nm _of 0.05 in the same buffer containing 20 mM glucose and 100 mM KCl. The cells were first treated with 15 mM EDTA pH 8.0 to permeabilize the outer membrane and allow the dye to reach the cytoplasmic membrane. Then, a stock solution of DiSC_3 _was added to a final concentration of 0.4 μM, and quenching was allowed to occur at room temperature. The desired concentration of peptides to be tested was added. Membrane depolarization was monitored with the Fluorescan Ascent FL microplate fluorometer by observing the change in the intensity of fluorescence (λ_ex _= 646 nm, λ_em _= 678 nm) after the addition of the peptides.

### Preparation of large unilamellar vesicles (liposomes) and leakage of calcein

Large unilamellar vesicles (liposomes) containing pure phosphatidylglycerol (PG) were prepared according to the previously described procedure [27]. Liposome-entrapped calcein and removal of free calcein by Sephadex G-50 chromatography were carried out essentially as described [65].

For the calcein release assay, 10 μL of liposome suspension were diluted in 10 mM Tris-HCl pH 7.4, 150 mM NaCl buffer (final vol of 100 μL) and incubated for 15 min at room temperature in the presence or absence (negative control) of the indicated peptides at 8 μM or in the presence of 1% Triton X-100 (positive control). The change in the intensity of fluorescence (λ_ex _= 485 nm, λ_em _= 527 nm) was monitored with a Fluorescan Ascent FL microplate fluorometer.

### Confocal microscopy

Bacteria were grown at 37°C with agitation in PSB medium to mid-logarithmic phase. Then, the cells were harvested by centrifugation, washed three times with 10 mM sodium phosphate buffer, pH 7.4 and resuspended in the same buffer to an A_600 _= 0.1. Bacteria were incubated with fluorescein-labeled full-length pre-elafin/trappin-2, prepared as described previously [27], for 1 h at 37°C in the dark. After incubation, cells were washed three times with phosphate buffer, and bacterial cells were mounted on a glass slide and microscopic observations (400 × magnification) of serial 0.2 μm sections were done with a Zeiss LSM 310 confocal microscope. Images were taken with an Olympus DP20 camera. As a negative control, free fluorescein incubated with bacteria and washed under the same conditions gave no fluorescent signal (data not shown).

### DNA binding assay

EMSA experiments were performed by mixing 100 ng of plasmid DNA (pRS426) with increasing amounts of recombinant peptides in 20 μl of binding buffer (5% glycerol, 10 mM Tris-HCl (pH 8.0), 1 mM EDTA, 1 mM DTT, 20 mM KCl and 5% (w/v) BSA). DNA samples with or without peptides were co-incubated at room temperature for 1 h prior to electrophoresis on a 1.0% agarose gel.

### Virulence factors assays

To assay for biofilm formation of *P. aeruginosa *an overnight culture was used to inoculate (~10^6 ^cells/ml) peptone soy broth media in 96 wells plates (Falcon 353072) in the presence or absence of recombinant peptide. The peptides were resuspended in 10 mM phosphate buffer (pH 7.4). The plate was incubated at 30°C for 26 h without agitation. The amounts of biofilm were determined by the method described by Peeters *et al. *[66] using the dye crystal violet. Alginate production of *P. aeruginosa *from a 24 h culture was assayed according to the procedure described by Pedersen *et al. *[67]. The enzymatic assay for lasB, from the cleared supernatants of a 24 h *P. aeruginosa *culture, was performed with the Congo red method as described previously [27]. The amounts of pyoverdine secreted by the bacteria were estimated by measuring the absorbance at 405 nm of the cleared culture supernatants from 24 h cultures of *P. aeruginosa *as described by Ambrosi *et al. *[68].

## Authors' contributions

AB carried out the purification of peptides, prepared the samples for CD, NMR and SEM analyses, analyzed the spectra for backbone assignments and secondary structures, performed the experiments on the release of liposome-entrapped calcein and the expression of virulence factors and participated in drafting the manuscript. NV carried out the membrane depolarization studies, the confocal microscopy examinations with fluorescein-labeled pre-elafin/trappin-2 and drafted the manuscript. SM analyzed NMR data and drafted the manuscript. SMG designed and analyzed NMR experiments. YB conceived the study, participated in its design and wrote the manuscript. All the authors have read and approved the final manuscript. The authors declare no competing interest.

## Supplementary Material

Additional file 1 **Supplementary_Figures**. **Fig. S1 - **Spin relaxation data (R_1_, R_2 _and NOE) and associated reduced spectral density mapping values. **Fig. S2 - **Diffusion behavior of cementoin, H_2_O and bicelles in different conditions. Click here for file
