# Supplementary material for: Structural and antimicrobial properties of human pre-elafin/trappin-2 and derived peptides against Pseudomonas aeruginosa
Source: BMC Microbiol. 2010 Oct 8;10:253. doi: 10.1186/1471-2180-10-253 (PMC2958999; doi:10.1186/1471-2180-10-253)
Supplement: Additional file 1 — Supplementary_Figures. Fig. S1 - Spin relaxation data (R1, R2 and NOE) and associated reduced spectral density mapping values. Fig. S2 - Diffusion behavior of cementoin, H2O and bicelles in different conditions. [file 1471-2180-10-253-S1.PDF]

## Supplementary material

# Structural and antimicrobial properties of human pre-elafin/trappin-2 and derived peptides against *Pseudomonas aeruginosa*


Audrey Bellemare, Nathalie Vernoux, Sébastien Morin, Stéphane M. Gagné and Yves Bourbonnais†

†From the Département de biochimie, microbiologie et bio-informatique, Institut de biologie intégrative et des systèmes and Centre de recherche PROTEO, Université Laval, Québec, Qc, Canada.


**Fig. S1** Spin relaxation data ($R_1$, $R_2$ and NOE) and associated reduced spectral density mapping values. (A) Spin relaxation data. (B) Reduced spectral density mapping values. (C) Correlation plots of J(0) with either $J(\omega_N)$ or $J(\omega_H)$. N-terminus residues (2-7) are shown with a X sign, C-terminus residues (33-38) with a + sign. These residues are the most flexible residues of cementoin and are located directly before and after predicted α-helices (from SSP and AGADIR). Residues 20 and 30 appear as outsiders, residue 20 probably experiencing slow μs-ms motions and residue 30, potentially high amplitude ps-ns motions.

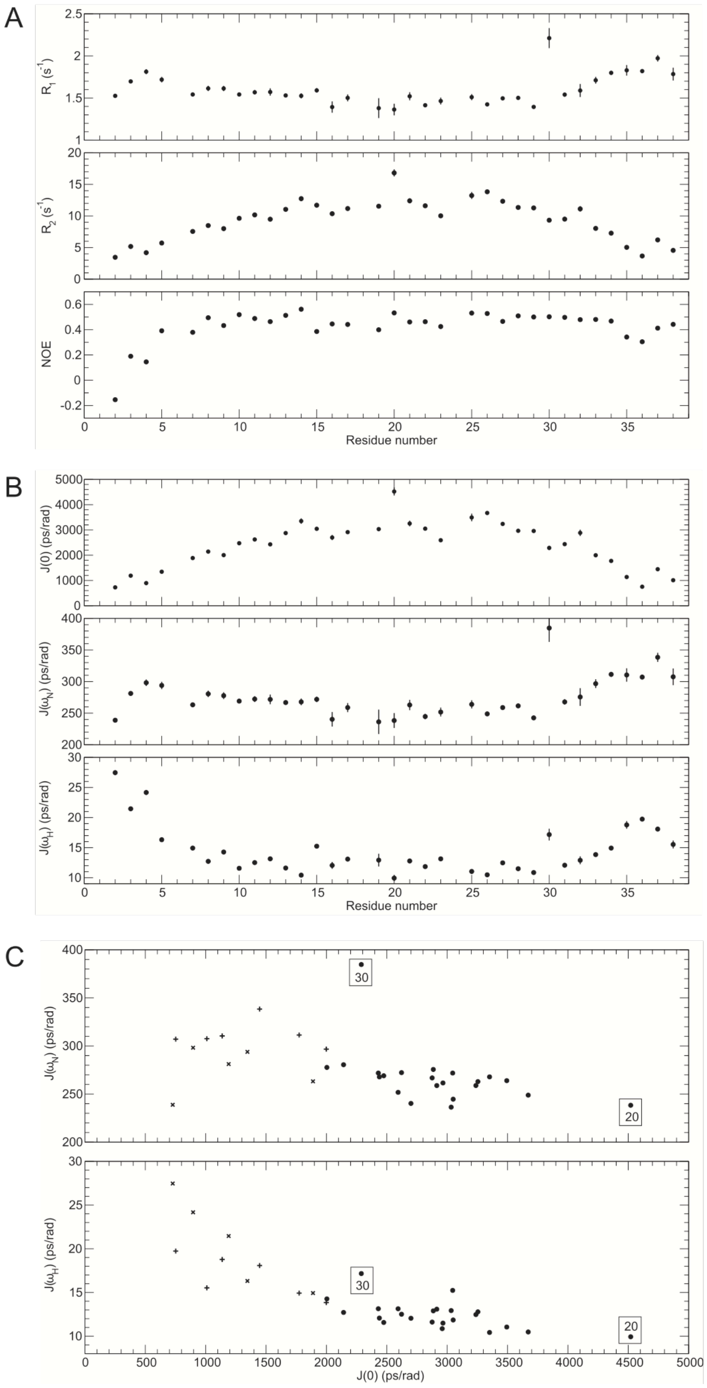

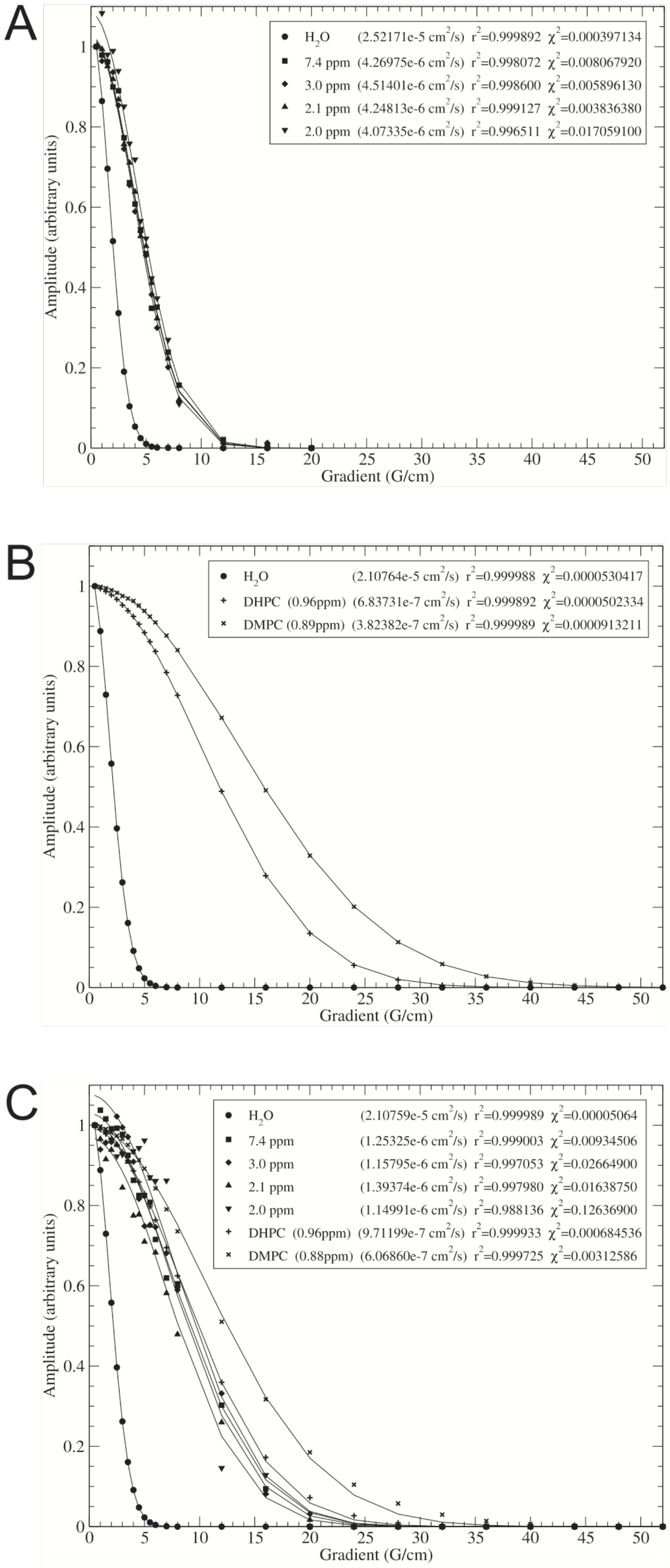


**Fig. S2** Diffusion behavior of cementoin, $H_2O$ and bicelles in different conditions. (A) Diffusion of $H_2O$ and cementoin in absence of bicelles. (B) Diffusion of $H_2O$ and bicelles in absence of cementoin. (C) Diffusion of $H_2O$, bicelles and cementoin in a mixed solution. Bicelles were composed of DHPC, DMPC and DMPG in the following ratio: 8:3:1.
